# Diagnosis of bovine viral diarrhea virus: an overview of currently available methods

**DOI:** 10.3389/fmicb.2024.1370050

**Published:** 2024-04-05

**Authors:** Yuting Wang, Feng Pang

**Affiliations:** Department of Veterinary Medicine, College of Animal Science, Guizhou University, Guiyang, China

**Keywords:** bovine viral diarrhea virus, diagnosis, etiological methods, serological methods, molecular methods, biosensors

## Abstract

Bovine viral diarrhea virus (BVDV) is the causative agent of bovine viral diarrhea (BVD), which results in significant economic losses in the global cattle industry. Fortunately, various diagnostic methods available for BVDV have been established. They include etiological methods, such as virus isolation (VI); serological methods, such as enzyme-linked immunosorbent assay (ELISA), immunofluorescence assay (IFA), and immunohistochemistry (IHC); molecular methods, such as reverse transcription-polymerase chain reaction (RT-PCR), real-time PCR, digital droplet PCR (ddPCR), loop-mediated isothermal amplification (LAMP), recombinase polymerase amplification (RPA), and CRISPR-Cas system; and biosensors. This review summarizes the current diagnostic methods for BVDV, discussing their advantages and disadvantages, and proposes future perspectives for the diagnosis of BVDV, with the intention of providing valuable guidance for effective diagnosis and control of BVD disease.

## 1 Introduction

Bovine viral diarrhea (BVD) is a highly infectious disease that causes significant economic losses in the global cattle industry. Bovine viral diarrhea virus (BVDV), the causative agent of BVD, belongs to the *Pestivirus* genus of the *Flaviviridae* family, which also includes classical swine fever virus (CSFV) and border disease virus (BDV) (Gao et al., 2011; Pang et al., 2023). Cattle of all breeds and ages are the natural host of BVDV, other animals such as goats, sheep, camels, pigs, and giraffes can also be infected. Infected animals exhibit persistent diarrhea, blood or mucus feces, obvious mucosal ulceration, reproductive disorders, and elevated body temperature (Tao et al., 2013; Rivas et al., 2022). BVDV can be classified into two biotypes, non-cytopathogenic (ncp) and cytopathogenic (cp), based on whether it produces a cytopathogenic effect (CPE) in infected cells (Merwaiss et al., 2019). Cp BVDV strains are not common and are generally involved in mucosal disease outbreaks, whereas ncp BVDV strains are more prevalent in nature and are often associated with severe acute infections (Oguejiofor et al., 2019). Pregnant females infected with BVDV can cause abortion, fetal death, or give birth to persistently infected (PI) animals. These PI animals remain infected throughout their lives and continuously shed the virus, posing a significant risk for BVDV transmission (Nugroho et al., 2022).

BVDV is a single-stranded, positive-sense RNA virus with a genome of approximately 12.3 kb. Its genome contains a single open reading frame (ORF) flanked by a 5′-untranslated region (5’-UTR) and 3’-UTR. While the 5’-UTR is commonly used for BVDV genotype and subtype classification (Mirosław and Polak, 2019), this method may lead to inaccurate or poorly statistically supported viral classification. Researchers have designed novel primer sets targeting NS3-NS4A of BVDV-1 (526 bp amplicon) and NS5B of BVDV-2 (728 bp amplicon) for subtyping BVDV. This classification accurately reproduces the subtyping of all the 118 BVDV-1 and 88 BVDV-2 complete/near-complete genomes (CNCGs) from GenBank (Mucellini et al., 2023). BVDV is classified into three genotypes, BVDV-1 (*Pestivirus* A), BVDV-2 (*Pestivirus* B), and BVDV-3 (*Pestivirus* H or HoBi-like virus), in which BVDV-1 currently contains at least 22 subtypes (1a-1v) and BVDV-2 contains at least 4 subtypes (2a-2d) (de Oliveira et al., 2021; Zhu et al., 2022). The single ORF encodes a large polyprotein that is post-translationally cleaved into four structural proteins (C, E^rns^, E1, E2) and eight non-structural proteins (N^pro^, p7, NS2, NS3, NS4A, NS4B, NS5A, NS5B) in ncp BVDV isolates (Chi et al., 2022). BVDV isolates, like other RNA viruses, exhibit high genetic variability, primarily due to recombination of non-homologous RNAs. Homologous RNA recombination also increases genetic diversity, which interferes with the diagnosis of BVDV and affects the efficacy of the BVDV vaccine (Zimmer et al., 2002; Fulton et al., 2003).

**Figure 1:**
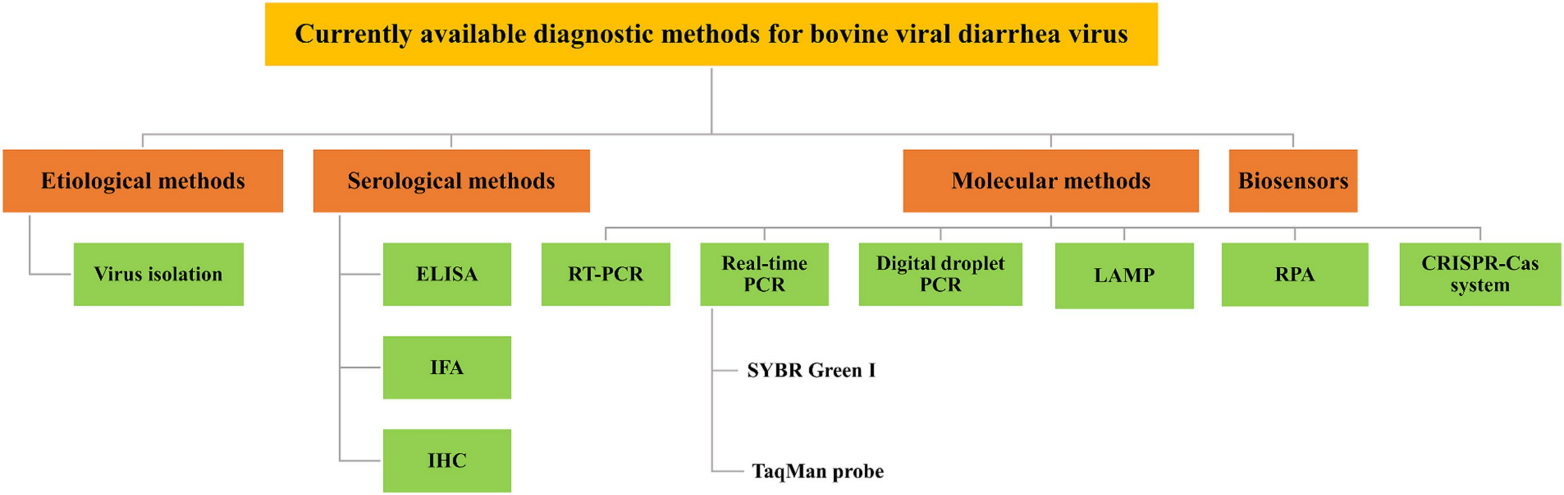
Schematic representation of currently available diagnostic methods for BVDV. Various diagnostic methods available for BVDV have been established, which include etiological methods, such as virus isolation (VI); serological methods, such as enzyme-linked immunosorbent assay (ELISA), immunofluorescence assay (IFA), and immunohistochemistry (IHC); molecular methods, such as reverse transcription-polymerase chain reaction (RT-PCR), real-time PCR, digital droplet PCR (ddPCR), loop-mediated isothermal amplification (LAMP), recombinase polymerase amplification (RPA), and CRISPR-Cas system; and biosensors.

BVD is a significant infectious disease that poses a threat to animal health and has caused a severe impact on the cattle industry (Qi et al., 2022). A rapid and accurate diagnosis of BVDV infection is crucial, and various diagnostic approaches have been established, including etiological, serological, and molecular methods for detecting BVDV. This review provides a comprehensive analysis of various BVDV detection methods, including their characteristics, advantages, and shortcomings, aiming to provide valuable support for the diagnosis, prevention and control of BVDV (Table 1). Figure 1 shows a schematic representation of the diagnostic methods currently available for the bovine viral diarrhea virus.

**Table 1:** Comparison of currently available diagnostic methods for BVDV.

Test	Target gene/protein	Sensitivity	Specificity	Positive rate	Turnaround time	Sample source in the literature	References
Indirect ELISA	Recombinant C-terminal truncated E2 protein	100% compared to VNT	98/99 (98.9%) compared to VNT	98/183 (54%)	No data	Serum samples	Marzocca et al. (2007)
Competitive ELISA	NS3 protein	93.90% compared to VNT	100% compared to VNT	77/197 (39%)	No data	Serum samples	Shapouri et al. (2022)
Multiplex indirect ELISA	E2 protein	100% compared to commercial ELISA kits*	94.7% compared to commercial ELISA kits*	No data	No data	Serum samples	Rodriguez et al. (2023)
IFA	E0 and E2 protein	No data	No data	No data	No data	E0 + E2 or E2 + E2 virus-like particles (VLPs)	Yang N. et al. (2022)
IHC	BVDV antigen	No data	No data	No data	No data	Oral mucosa and skin samples	Bianchi et al. (2017)
RT-PCR	5’-UTR	No data	No data	No data	< 4 h	Organs (lungs, intestines, brains) or leukocytes	Letellier et al. (1999)
Multiplex RT-PCR	5’-UTR	30 TCID_50_/mL	No data	6/22 (27%)	< 4 h	Oral and nasal materials	Lung et al. (2017)
SYBR Green I real-time PCR	NS5B gene	100 copies/mL	No primer-dimers and non-specific products, only a single peak in the melt curve plot	No data	No data	Viral RNA	Zhang et al. (2011)
SYBR Green I real-time PCR	5’-UTR	5.2 RNA molecules per reaction	No cross-reaction with CSFV, BDV, BVDV-2, IBRV, BPIV-3, BRSV, BEFV, and BcoV	29/169 (17.2%)	No data	Aerosol samples	Hou et al. (2020)
TaqMan real-time PCR	5’-UTR and 3’-UTR	1.55 copies/μL	No cross-reaction with JEV, CSFV, RABV, BRV, BPV, and FMDV	49/312 (16%)	No data	Feces samples	Liang et al. (2019)
Multiplex TaqMan real-time PCR	5’-UTR	3.2 TCID_50_	No cross-reaction with HCLV, BDV, PRRSV, PCV, PPV, PRV, PEDV, and TGEV	24/176 (13.6%)	No data	Serum samples, lymph nodes, spleens, and tonsils	Zhang et al. (2012)
ddPCR	Viral RNA	13 copies/μL	No data	No data	No data	Fetal bovine serum	Henrique et al. (2023)
RT-LAMP	5’-UTR	4.67 RNA copies	No cross-reaction with BRV, *Mycobacterium bovis*, CSFV, BoHV-1, and BCV	38/88 (43%)	60 min	Fecal swabs	Fan et al. (2012)
RT-RPA	5’-UTR	50 copies/μL	No cross-reaction with BCoV, CSFV, BRSV, IBRV, and BRV	36/48 (75%)	25 min	Blood samples and nasal swab	Yang S. et al. (2022)
CRISPR-Cas13 system	5’-UTR	0.2 μM	No data	No data	No data	Synthetic RNA/ *in vitro* viral RNA transcripts	Hwang et al. (2023)
CRISPR-Cas13 system	5’-UTR	10^3^ pM	No cross-reaction with HEK293T and MDBK	No data	No data	Synthetic RNA/ *in vitro* viral RNA transcripts	Yao et al. (2021)
Electrochemical biosensor system	E^rns^ gene	10^3^ CCID/mL	No data	No data	8 min	Serum samples	Luo et al. (2010)
Electrochemical biosensor system	5’-UTR	Cross-linking (CL): 6.83 ng/reaction; non-crosslinking (NCL): 44.36 ng/reaction	100% for CL method and 97% for NCL compared to RT-nested multiplex PCR and RT real-time PCR	CL: 18/50 (36%) NCL: 17/50 (34%)	CL: 20 min NCL:40 min	Serum samples	Heidari et al. (2021)
Electrochemical biosensor system	Viral RNA	0.59 copies/mL	less affected by interferents such as BCV, BRV, DeV and NoV	No data	10 min	No data	Kim et al. (2023)

VNT, virus neutralization test; CSFV, classical swine fever virus; BDV, border disease virus; IBRV, infectious bovine rhinotracheitis virus; BPIV-3, bovine parainfluenza virus type 3; BRSV, bovine respiratory syncytial virus; BEFV, bovine ephemeral fever virus; BcoV/BCV, bovine coronavirus; JEV, Japanese encephalitis virus; RABV, rabies virus; BRV, bovine rotavirus; BPV, bovine parvovirus; FMDV, foot and mouth disease virus; HCLV, hog cholera virus; PRRSV, porcine reproductive and respiratory syndrome; PCV, porcine circovirus; PPV, porcine parvovirus; PRV, pseudorabies virus; PEDV, porcine epidemic diarrhea virus; TGEV, transmissible gastroenteritis virus; MDBK, Madin-Darby bovine kidney; BoHV-1, bovine-herpesvirus 1; DeV, denguevirus; NoV, norovirus. *Commercial ELISA monoplex kits (BVDV 994400; IBR Ab 99–41,459; PI3 P00652-2; BRSV P00651-2; EBLV; P02110-5; Idexx Laboratories, Westbrook, Maine, USA).

## 2 Etiological methods for the diagnosis of BVDV

### 2.1 Virus isolation

Virus isolation (VI) is the most reliable method for detecting BVDV and is considered the “gold standard” for BVDV diagnosis (Sandvik, 2005). Primary cells such as bovine uterine endometrial cells (Cheng et al., 2017), bovine testis (BT) cells (Weber et al., 2017), bovine kidney (BK) cells (Suda et al., 2019), bovine turbinate (BTu) cells (Falkenberg et al., 2021), bovine bronchial epithelial (BBE) cells (Su et al., 2021), bovine lung (BL) cells (La Polla et al., 2022a,b), and bovine skin fibroblast (BSF) cells (Workman et al., 2023) can be used for the isolation of BVDV. Among these, BK, BT, and BTu cells are currently the most widely used primary cells for BVDV isolation. In addition, BVDV isolates can be cultured *in vitro* in Madin-Darby bovine kidney (MDBK), bovine tonsil (BoTur), and baby hamster kidney (BHK) cell lines. MDBK is more susceptible to BVDV infection (Odeón et al., 2009), and is commonly used for BVDV culture (Workman et al., 2021; Yang et al., 2021; Wang et al., 2023).

The VI assay is highly accurate and does not require complex instruments, but some problems should not be underestimated. First, virus isolation can only identify cp BVDV, but not ncp BVDV. Second, this method is labor intensive, time-consuming, and not suitable for processing a large number of samples. Third, the final judgment of BVDV infection tends to be influenced by several factors, such as the sensitivity of inoculated cells and the way the sample is preserved or transported.

## 3 Serological methods for the diagnosis of BVDV

### 3.1 Enzyme-linked immunosorbent assay

Enzyme-linked immunosorbent assay (ELISA) is a rapid and high-throughput serological diagnostic method for the detection of a specific antigen or antibody from certain pathogens. The main types of ELISA include direct, indirect, double-antibody sandwich, and competitive methods (Tabatabaei and Ahmed, 2022). Zhang et al. used a purified recombinant BVDV-E2 protein to immunize chickens and acquired the specific E2-IgY antibody from egg yolk. A total of 22 blood samples from diarrheic cattle were collected to assess the indirect ELISA assay based on E2 IgY and the assay yielded 95.45% concordance with the RT-PCR assay for detecting BVDV (Zhang et al., 2016). Furthermore, Marzocca et al. developed an indirect ELISA for the detection of BVDV using a recombinant C-terminal truncated E2 protein (tE2) expressed in the *Drosophila melanogaster* system. The tE2 protein was efficiently secreted in the supernatant in the form of post-translational modification, without the need for purification. The initial examination of 183 cattle serum samples utilizing the tE2-ELISA demonstrated a specificity of 98% and a sensitivity of 100% compared to the standard BVDV neutralization test (Marzocca et al., 2007). Shapouri et al. developed a competitive ELISA (cELISA) for detecting BVDV antibodies in serum samples. The NS3 protein, which was highly conserved among pestiviruses, was used as a recombinant antigen in combination with a monoclonal antibody as a competitive antibody. The cELISA assay was used to analyze a total of 197 serum samples, demonstrating a sensitivity of 93.90% and a specificity of 100% when compared to the virus neutralization test (Shapouri et al., 2022). Moreover, a multiplex indirect ELISA assay was developed to identify antibodies against five viral pathogens, including bovine respiratory syncytial virus (BRSV), bovine herpes virus-1 (BoHV-1), bovine viral diarrhea virus (BVDV), parainfluenza virus type 3 (PI3V), and enzootic bovine leukosis virus (EBLV) using complete viruses of BRSV and BoHV-1, and recombinant E2/BVDV, HN/PI3V, and gp51/EBLV as capture antigens. Diagnostic agreement for samples that were analyzed concurrently using monoplex and multiplex assays was nearly perfect for BoHV-1, EBLV, BRSV, and BVDV (above 0.81) and substantial for PI3V (ranging from 0.61 to 0.80) (Rodriguez et al., 2023). In summary, ELISA is the most commonly used technique in serology due to its high specificity, high sensitivity, good reproducibility, low cost, and ease of operation, making it a ideal choice for high-throughput applications.

### 3.2 Immunofluorescence assay

Immunofluorescence assay (IFA) is a technique in which a fluorescent antibody or antigen is used as a probe to detect an unknown antigen or antibody (Piña et al., 2022). Bedeković et al. developed an IFA method for the diagnosis of BVDV in which a specific monoclonal antibody for BVDV was used as primary antibody and an anti-mouse antibody conjugated with fluorescein isothiocyanate (FITC) as secondary antibody. The IFA assay showed a sensitivity and specificity of 100% in detecting 9 positive samples when compared to the RT-PCR assay (Bedeković et al., 2011). Yang et al. generated BVDV E0 + E2 or E2 + E2 virus-like particles (VLPs) using an insect baculovirus expression vector system. The expression of E0 + E2 and E2 + E2 were subsequently detected by immunofluorescence assay (IFA) and western blot (Yang N. et al., 2022). In another experiment, the IFA was applied to detect a positive BVDV-3 strain using BVDV-positive serum as primary antibody and FITC-labeled rabbit anti-bovine IgG as secondary antibody. MDBK cells infected with the BVDV-3 strain were observed to exhibit green fluorescence while uninfected cells did not (Yang et al., 2023). IFA has the advantages of good sensitivity and specificity, but non-specific staining phenomenon occurs, and it needs to be observed with the help of a special fluorescence microscope, which is costly and unsuitable for the promotion of the application at the grass-roots level.

### 3.3 Immunohistochemistry

Immunohistochemistry (IHC) is a technique of detecting antigen in tissues by a chemical reaction that results in the coloration of a chromogenic agent based on the binding of antibodies to a specific antigen in tissue sections (Harms et al., 2023). In a previous study, a total of 184 cattle underwent immunohistochemical tests on skin biopsies to determine the presence of BVDV infection. The IHC assay was determined to be sensitive and specific in detecting BVDV infection (Thür et al., 1996). Bianchi et al. used oral mucosa, skin tissue, and small intestine sections from three calves that died from BVDV infection for the IHC assay. The results showed notable immunostaining within the cytoplasm of epidermal and follicular epithelial cells, indicating the presence of the BVDV antigen within the cytoplasm of skin and mucosal epithelial cells (Bianchi et al., 2017). The IHC assay is sensitive, rapid, and can be applied to a large number of samples. Furthermore, the method is not interfered by maternal antibodies in blood samples and is therefore widely used in the detection of PI animals (Brodersen, 2004; Hilbe et al., 2007). However, IHC is a labor intensive test that is prone to operational problems and has more subjective influences on the criteria for judging the results, and it requires higher technical experience from personnel (Cornish et al., 2005).

## 4 Molecular methods for the diagnosis of BVDV

### 4.1 Reverse transcription-polymerase chain reaction

Reverse transcription-polymerase chain reaction (RT-PCR) involves extracting RNA from tissue cells, reverse transcribing it into complementary DNA (cDNA) using reverse transcriptase, and finally using cDNA as a template for PCR amplification (Donovan et al., 2022). The RT-PCR method for detecting BVDV was first established by Hertig et al. in 1991, based on the p80 and gp53 genes that encode the NS3 and E2 protein, respectively (Hertig et al., 1991). Since then, this method has been widely used for the diagnosis of BVDV. Letellier et al. developed an RT-PCR assay based on the highly conserved 5’-UTR region to detect both the BVDV-1 and BVDV-2 genotypes and differentiate them from other plague viruses (Letellier et al., 1999). Monteiro et al. designed and evaluated various primer sets to detect 135 serum samples positive for BVDV antigens by an antigen ELISA using RT-PCR assays. Compared to other primer sets, the newly designed BP189-389 primers had the ability to detect all 135 ELISA-positive samples including BVDV-1 (*n* = 64), BVDV-2 (*n* = 45), and HoBi-like pestivirus (*n* = 26) (Monteiro et al., 2019). Furthermore, several multiplex RT-PCR approaches have been established and utilized for BVDV detection, in addition to single-gene RT-PCR. Lung et al. have developed a highly accurate multiplex RT-PCR and an innovative automated microarray that can simultaneously detect eight viruses that affect cattle, including BVDV-1 and BVDV-2, vesicular stomatitis virus (VSV), bluetongue virus (BTV), malignant catarrhal fever virus (MCFV), bovine herpesvirus-1 (BoHV-1), parapoxvirus (PPV), and rinderpest virus (RPV). The approach accurately identified a panel of 37 strains of the eight target viruses and detected a mixed infection. Limit of detection of the multiplex RT-PCR and microarray assay for BVDV were both 30 TCID_50_/mL (Lung et al., 2017). RT-PCR is a superior method of detection, offering faster detection, greater specificity, and higher sensitivity compared to virus isolation and IFA. In addition, RT-PCR can determine the nucleic acid sequence of BVDV in samples in which the virus has been inactivated.

### 4.2 Real-time PCR

Real-time PCR is an appealing alternative to conventional PCR since it can monitor the production of amplification products in real-time through the detection of a fluorescent signal during each cycle of the PCR reaction. Real-time PCR assays are highly specific, sensitive, and highly automated, making them ideal for high-throughput measurements (Bustin, 2005). The SYBR Green I dye and the TaqMan probe are the two most commonly used real-time PCR assays. TaqMan-based real-time PCR is significantly more specific for nucleic acid detection and quantification compared to the SYBR Green I assay, although the cost of synthesizing specific probes is too high for grassroots use.

#### 4.2.1 SYBR Green I real-time PCR

In 2006, Young et al. developed a highly sensitive two-step SYBR Green I real-time RT-PCR assay to detect acute BVDV infection in whole blood from cattle. The assay targets a conserved region of the BVDV 5’-UTR and can detect samples containing as low as 2.1 × 10^1^ TCID_50_ BVDV (Young et al., 2006). Additionally, Zhang et al. established a one-step SYBR Green I real-time PCR assay based on NS5B for the detection of BVDV-1 in cell culture. The assay had a detection limit as low as 100 copies/mL of BVDV RNA and a maximum intra-assay CV of 2.63%. Its sensitivity was found to be ten times higher than that of conventional RT-PCR, enabling quantitative detection of BVDV RNA levels across a range of ten-fold serial dilutions of titrated viruses with titers ranging from 10^−1^ to 10^−5^ TCID_50_ without any nonspecific amplification (Zhang et al., 2011). Hou et al. developed a highly specific SYBR Green I real-time PCR assay based on the highly conserved regions of the 5’-UTR for the quantitative detection of BVDV-1 in aerosol samples. Importantly, the assay did not cross-react with other common infectious bovine viral diseases such as CSFV, BDV, and BVDV-2, demonstrating its reliability and accuracy. The lowest detection limit was 5.2 RNA molecules per reaction. Compared to conventional RT-PCR, the assay exhibited a significantly higher positive detection rate (17.2%, 29/169) when used to test a total of 169 aerosol samples collected from six dairy herds. Furthermore, a concordance rate of 100% was achieved between the assay and the BVDV RPA-LFD assay in detecting the positive samples (Hou et al., 2020). An interesting study reported that the SYBR Green I real-time RT-PCR targeting 5’-UTR and nested RT-PCR are superior to antigen-capture ELISA (Ag ELISA) for BVDV detection in aborted fetus samples over a 22-year period (Spetter et al., 2020).

#### 4.2.2 TaqMan real-time PCR

Baxi et al. developed a highly accurate one-step multiplex real-time PCR assay using SmartCycler technology and type-specific TaqMan probes for the detection of BVDV-1 and BVDV-2. The assay successfully typed 54 BVDV strains and field isolates, demonstrating the high specificity of the TaqMan probe without any reactivity to CSFV and BDV (Baxi et al., 2006). Liang et al. developed a highly sensitive TaqMan real-time PCR method for BVDV detection, with a detection limit of 1.55 copies/μL for viral RNA. This is a significant improvement over traditional RT-PCR, with a 10,000-fold increase in sensitivity. Notably, the assay demonstrated no cross-reactivity with other viruses, including CSFV, BRV, JEV, RABV, BPV, and FMDV. The assay and conventional RT-PCR were used to detect 312 feces samples harvested from diarrhea calves from six cattle farms. The study unequivocally demonstrates the superiority of the established method, which produced positive results in 49 feces samples, compared to the universal PCR method that only detected positive results in 44 samples (Liang et al., 2019). Zhang et al. established a highly sensitive triple TaqMan real-time PCR assay capable of detecting BVDV-1, wild-type CSFV, and hog cholera laminated vaccine (HCLV), with a detection limit of 3.2 TCID_50_ for BVDV-1, 4.5 TCID_50_ for CSFV, and 10 TCID_50_ for HCLV. The method detects three targets simultaneously and without mutual interference, making it a highly sensitive tool for the simultaneous detection and differentiation of BVDV-1, CSFV, and HCLV (Zhang et al., 2012). Furthermore, a one-step multiplex TaqMan real-time PCR assay was developed that can simultaneously detect all five pathogens responsible for the bovine respiratory disease complex (BRDC): BVDV, BoHV-1, bovine parainfluenza virus type 3 (BPIV-3), bovine respiratory syncytial virus (BRSV), and influenza D virus (IDV). The method confidently detects BRDC-related pathogens from bovine nasal swabs with signs of respiratory disease, and the detection limits of the five pathogens were 10^2^ copies/μL (Zhang et al., 2022). In a recent study, researchers initiated an international interlaboratory proficiency trial to evaluate different diagnostic methods for BVDV using a sample panel of four ear notch samples and four sera in 40 veterinary diagnostic laboratories from 10 countries. They found that various RT-qPCR assays or E^rns^ antigen ELISA were highly recommended due to their superior diagnostic sensitivity compared to virus isolation for the identification of BVDV in clinical cases (Wernike and Beer, 2024).

### 4.3 Digital droplet PCR

Digital droplet PCR (ddPCR) is a powerful technique that combines PCR with droplet microfluidics, allowing for the precise quantification of target molecules in a sample. By dropletising the sample before amplification, ddPCR enables the absolute quantification of nucleic acid molecules with unparalleled accuracy and sensitivity (Chen et al., 2021). Flatschart et al. successfully detected BVDV genome copies in cell cultures using ddPCR, demonstrating its potential as a reliable and robust method for nucleic acid analysis. This assay enables the absolute quantification of the BVDV genome without the use of calibration standards (Flatschart et al., 2015). In contrast, ddPCR confidently achieved a limit of detection of 13 copies/μL and a limit of quantification of 38 copies/μL for BVDV, demonstrating its superiority in absolute quantitative studies of BVDV RNA (Henrique et al., 2023). The ddPCR offers significant technical advantages and has promising applications, making it a reliable choice for researchers. First, it allows for absolute quantification of target molecules without the need for standards. Second, it is fast, accurate and effectively avoids false positives and false negatives. Third, the technique is not dependent on amplification efficiency and is particularly suitable for detecting samples with complex matrices. Finally, the results are easy to analyze.

### 4.4 Loop-mediated isothermal amplification

Loop-mediated isothermal amplification (LAMP) is a rapid thermostatic nucleic acid amplification technique developed by Notomi in 2000 for the detection of various infectious diseases (Notomi et al., 2000; Pang and Long, 2023). This multistep reaction is carried out at around 60–70°C using strand-displacing DNA polymerase and 4–6 primers that recognize 6–8 unique sites on the target nucleic acid.

Aebischer et al. developed three highly sensitive assays, including high-speed RT-qPCR, LAMP, and RPA and compared their performance for detecting Schmallenberg virus and BVDV (Aebischer et al., 2014). Furthermore, Fan et al. optimized a reverse transcription loop-mediated isothermal amplification (RT-LAMP) method for detecting BVDV-1 and BVDV-2 using six primer pairs that were designed based on the 5’-UTR of the BVDV genome. The method was able to detect 4.67 RNA copies without the need for complex equipment. In addition, it has high specificity and no cross-reactivity with BRV, CSFV, BoHV-1, and BCoV. This method holds great promise for clinical diagnosis and field monitoring of BVDV (Fan et al., 2012). LAMP amplification products are commonly identified through agarose gel electrophoresis. The incorporation of fluorescent dyes into the LAMP product allows for direct visual detection of positive products. In the study conducted by Tajbakhsh et al., GelRed fluorescent dye was used to detect LAMP-amplified BVDV products. Positive samples were observed to have a fluorescent yellow reaction color, while negative samples retained their red reaction color under UV light. This method is advantageous in terms of simplicity, time-saving, and cost-effectiveness, making it suitable for detecting BVDV in the field (Tajbakhsh et al., 2017). However, it should be noted that LAMP is not suitable for detecting multiple pathogens as it cannot accurately identify the effect of a single pathogen among multiple pathogens due to its single expression (Ayaz Kök et al., 2023).

### 4.5 Recombinase polymerase amplification

Recombinase polymerase amplification (RPA) is an isothermal amplification system that is highly effective for virus detection, especially for point-of-care testing (POCT) (Munawar, 2022). The RPA reaction uses recombinant enzymes and oligonucleotide primers to form protein-DNA complexes that efficiently search for homologous sequences in double-stranded DNA. By initiating a strand displacement reaction, it rapidly initiates DNA synthesis that exponentially amplifies the target on the template. The single-stranded DNA binding protein (SSB) binds to the replaced DNA strand and forms a D-loop that prevents further substitutions (Li and Macdonald, 2015). The whole amplification process is rapid and reaches detectable levels within minutes. Yang et al. developed a method for the rapid and easy detection of BVDV and BPIV-3 by targeting the BVDV 5’-UTR and the phosphorylated protein P gene of BPIV-3, through the combined application of reverse transcriptase recombinase polymerase amplification (RT-RPA) and lateral flow test paper (LFD). The method amplifies BVDV and BPIV3 RNA by applying RT-RPA within 25 min at 35°C. The reaction products are observed on the LFD within just 5 min at room temperature. The lowest detection limit of 50 copies/μL for BVDV and 34 copies/μL for BPIV-3 further showcase the impressive capabilities of this method. Notably, the method does not cross-react with viruses such as CSFV and IBRV (Yang S. et al., 2022). The RPA technology exhibits a notable level of specificity and sensitivity, undergoes rapid amplification, offers ease of operation, and requires a lower temperature than RT-PCR and LAMP, making it a highly efficient option for virus detection. Nevertheless, the RPA method has limitations, such as the potential for false positives during the detection process (Munawar, 2022).

### 4.6 CRISPR-Cas system

Clustered regularly interspaced short palindromic repeats (CRISPR)-CRISPR-associated proteins (Cas) systems have been widely used for genome editing due to its precise recognition and cleavage of specific DNA and RNA sequences. Furthermore, it is important to note that certain CRISPR/Cas systems, including Cas13, Cas12a, and Cas14, possess collateral nonspecific catalytic activities that can be harnessed for nucleic acid detection. This is accomplished by breaking down a labeled nucleic acid to generate a fluorescent signal (Aman et al., 2020).

Hwang et al. developed a highly efficient CRISPR-Cas13 system for point-of-care testing (POCT) of BVDV by precisely targeting the BVDV-1b 5’-UTR. Four specially designed and synthesized CRISPR RNAs (crRNAs) were utilized to ensure accurate targeting of the BVDV-1b 5’-UTR. The expression and purification of the LwCas13a protein was optimized and the RNase activity of LwCas13a was verified. The collateral cleavage activity of Cas13 was then confirmed using a reliable colorimetric lateral-flow detection assay (LFDA) (Hwang et al., 2023). Yao et al. also established a CRISPR-Cas13a system that successfully detected BVDV nucleic acid with a detection limit of 10^3^ picomolar (pM) using a fluorescence (FAM)- and quencher (BHQ-1)-labeled RNA probe (Yao et al., 2021). The CRISPR-Cas13a system-based nucleic acid detection method for BVDV offers several advantages. These include rapid detection, high sensitivity, good specificity, high-throughput, result visualization, and adaptability to point-of-care testing.

## 5 Biosensors

Biosensors combine a sensing element that detects target molecules and a transducer that converts a biochemical event into a measurable signal. The transducers can be electrochemical, optical, piezoelectric, acoustics, or calorimetric (Metkar and Girigoswami, 2019). Biosensors are a highly effective and widely used technique for detecting a range of infectious disease (Abid et al., 2021). Luo et al. successfully optimized an electrostatically spun capture membrane-based biosensor for the rapid quantitative detection of both *Escherichia coli* O157: H7 and BVDV with detection limits of 61 CFU/mL and 10^3^ CCID/mL, respectively, within just eight minutes (Luo et al., 2010). Heidari et al. developed two highly effective colorimetric biosensor assays based on probe-modified gold nanoparticles (AuNPs) to detect BVDV. Specific probes targeting the 5’-UTR of BVDV were immobilized on the surface of the AuNPs. The hybridization of positive targets with the probe-AuNPs resulted in the formation of a polymeric network among AuNPs, ultimately leading to the aggregation of nanoparticles and color change from red to blue. The cross-linking (CL) and non-crosslinking (NCL) probe-AuNPs assays demonstrated detection limits of 6.83 and 44.36 ng/reaction, respectively (Heidari et al., 2021). Kim et al. developed an electrochemical biosensor system for the rapid BVDV detection using synthetic conductive nanomaterials black phosphorus (BP) and AuNPs. AuNPs were synthesized on the surface of BP to enhance the conductivity. In addition, a dopamine-modified self-polymerization system was synthesized on the BP nanosurface to further enhance the stability of the biosensor and promote the immobilization of the affinity peptide. The system confidently detected BVDV at a minimum detection limit of 0.59 copies/mL within 10 min, and maintained electroactivity above 95% of its initial performance for 30 days. Notably, there was no cross-reactivity with bovine coronavirus (BCV), bovine rotavirus (BRV), dengue virus (DeV), and norovirus (NoV) (Kim et al., 2023). In another study, a paper strip method was developed for the rapid identification of BVDV through the utilization of BVDV-specific peptides with a high affinity for the virus and creating a copper polyhedral (CuP) nanoshell on the surface of AuNPs. The peptide-based optical biosensor demonstrated superior efficacy with a lower detection limit of 4.4 copies per mL, while the CuP nanoshell facilitated quantitative diagnosis of BVDV through the visual detection of a pink dot observable to the naked eye (Kim et al., 2020). In conclusion, biosensors have the advantages of portability, direct real-time detection, and fast response time, and have great potential for point-of-care testing of BVDV.

## 6 Conclusions and perspectives

BVDV can be detected through various methods. Virus isolation is considered the gold standard, despite being time-consuming and labor intensive. ELISA is also a commonly used serological detection method, thanks to its ease of operation and adaptability to the detection of a large number of samples. Molecular diagnostic methods such as RT-PCR, and real-time PCR, are widely used due to their higher specificity and sensitivity. Isothermal amplification techniques, such as LAMP and RPA, are highly suitable for the clinical detection of BVDV at the grassroots level due to their rapid and efficient detection, high specificity, and good sensitivity. Furthermore, techniques such as ddPCR and the CRISPR-Cas system, demonstrate significant potential application for the diagnosis of BVDV.

Accurate BVDV diagnosis and removal of persistently infected (PI) cattle are fundamental for controlling and eradicating the disease from herds. However, existing diagnostic methods for BVDV have varying levels of sensitivity and specificity, which can lead to misdiagnosis or missed infections due to false negative or false positive results. Combining different BVDV diagnostic methods will improve detection accuracy. Meanwhile, it is quite necessary for the development of faster, more accurate and more specific diagnostics, such as Next-Generation Sequencing (NGS), which can provide comprehensive genomic information about BVDV strains, aiding in understanding viral evolution and epidemiology. Vaccination remains an essential strategy for BVDV control in most countries. It is important to note that vaccinated animals can still test positive for BVDV antibodies. DIVA (differentiating infected from vaccinated animals) vaccines and accompanying diagnostic tests are needed for the eradication of BVDV in endemic countries.

## Author contributions

YW: Writing – original draft, Writing – review & editing. FP: Conceptualization, Funding acquisition, Visualization, Writing – original draft, Writing – review & editing.

## References

[ref1] Abid S. A. Muneer A. A. Al-Kadmy I. M. Sattar A. A. Beshbishy A. M. Batiha G. E.-S.. (2021). Biosensors as a future diagnostic approach for COVID-19. Life Sci. 273:119117. doi: 10.1016/j.lfs.2021.119117, PMID: 33508293 PMC7834970

[ref2] Aebischer A. Wernike K. Hoffmann B. Beer M. (2014). Rapid genome detection of Schmallenberg virus and bovine viral diarrhea virus by use of isothermal amplification methods and high-speed real-time reverse transcriptase PCR. J. Clin. Microbiol. 52, 1883–1892. doi: 10.1128/JCM.00167-14, PMID: 24648561 PMC4042763

[ref3] Aman R. Mahas A. Mahfouz M. (2020). Nucleic acid detection using CRISPR/Cas biosensing technologies. ACS Synth. Biol. 9, 1226–1233. doi: 10.1021/acssynbio.9b00507 32159950

[ref4] Ayaz Kök S. Üstün S. Taşkent Sezgin H. (2023). Diagnosis of ruminant viral diseases with loop-mediated isothermal amplification. Mol. Biotechnol. 65, 1228–1241. doi: 10.1007/s12033-023-00674-6, PMID: 36719638 PMC9888337

[ref5] Baxi M. McRae D. Baxi S. Greiser-Wilke I. Vilcek S. Amoako K.. (2006). A one-step multiplex real-time RT-PCR for detection and typing of bovine viral diarrhea viruses. Vet. Microbiol. 116, 37–44. doi: 10.1016/j.vetmic.2006.03.026, PMID: 16687219

[ref6] Bedeković T. Lemo N. Lojkić I. Cvetnić Ž. Čač Ž. Madić J. (2011). Development of an indirect immunofluorescence assay for diagnosis of bovine viral diarrhoea virus on ear notch tissue samples in cattle infected persistently. J. Virol. Methods 178, 59–62. doi: 10.1016/j.jviromet.2011.08.012, PMID: 21875618

[ref7] Bianchi M. Konradt G. De Souza S. Bassuino D. Silveira S. Mósena A.. (2017). Natural outbreak of BVDV-1d–induced mucosal disease lacking intestinal lesions. Vet. Pathol. 54, 242–248. doi: 10.1177/0300985816666610, PMID: 27586238

[ref8] Brodersen B. W. (2004). Immunohistochemistry used as a screening method for persistent bovine viral diarrhea virus infection. Vet. Clin. 20, 85–93. doi: 10.1016/j.cvfa.2003.11.007, PMID: 15062476

[ref9] Bustin S. A. (2005). Real-time, fluorescence-based quantitative PCR: a snapshot of current procedures and preferences. Expert. Rev. Mol. Diagn. 5, 493–498. doi: 10.1586/14737159.5.4.493, PMID: 16013967

[ref10] Chen B. Jiang Y. Cao X. Liu C. Zhang N. Shi D. (2021). Droplet digital PCR as an emerging tool in detecting pathogens nucleic acids in infectious diseases. Clin. Chim. Acta 517, 156–161. doi: 10.1016/j.cca.2021.02.008, PMID: 33662358

[ref11] Cheng Z. Chauhan L. Barry A. T. Abudureyimu A. Oguejiofor C. F. Chen X.. (2017). Acute bovine viral diarrhea virus infection inhibits expression of interferon tau-stimulated genes in bovine endometrium. Biol. Reprod. 96, 1142–1153. doi: 10.1093/biolre/iox056, PMID: 28605413

[ref12] Chi S. Chen S. Jia W. He Y. Ren L. Wang X. (2022). Non-structural proteins of bovine viral diarrhea virus. Virus Genes 58, 491–500. doi: 10.1007/s11262-022-01914-8, PMID: 35614328 PMC9131992

[ref13] Cornish T. E. van Olphen A. L. Cavender J. L. Edwards J. M. Jaeger P. T. Vieyra L. L.. (2005). Comparison of ear notch immunohistochemistry, ear notch antigen-capture ELISA, and buffy coat virus isolation for detection of calves persistently infected with bovine viral diarrhea virus. J. Vet. Diagn. Invest. 17, 110–117. doi: 10.1177/104063870501700203, PMID: 15825490

[ref14] de Oliveira P. S. B. Júnior J. V. J. S. Weiblen R. Flores E. F. (2021). Subtyping bovine viral diarrhea virus (BVDV): which viral gene to choose? *Infection*. Genet. Evol. 92:104891. doi: 10.1016/j.meegid.2021.104891, PMID: 33945882

[ref15] Donovan N. J. Chambers G. A. Cao M. (2022). Detection of Viroids by RT-PCR. Methods Mol. Biol. 2316, 143–151. doi: 10.1007/978-1-0716-1464-8_13 34845692

[ref16] Falkenberg S. M. Dassanayake R. P. Terhaar B. Ridpath J. F. Neill J. D. Roth J. A. (2021). Evaluation of antigenic comparisons among BVDV isolates as it relates to humoral and cell mediated responses. Front. Vet. Sci. 8:685114. doi: 10.3389/fvets.2021.685114, PMID: 34212022 PMC8239304

[ref17] Fan Q. Xie Z. Xie L. Liu J. Pang Y. Deng X.. (2012). A reverse transcription loop-mediated isothermal amplification method for rapid detection of bovine viral diarrhea virus. J. Virol. Methods 186, 43–48. doi: 10.1016/j.jviromet.2012.08.007, PMID: 22947692 PMC7112856

[ref18] Flatschart R. Almeida D. Heinemann M. Medeiros M. Granjeiro J. Folgueras-Flatschart A. (2015). Absolute quantification of bovine viral diarrhea virus (BVDV) RNA by the digital PCR technique. J. Phys. 575:12038. doi: 10.1088/1742-6596/575/1/012038

[ref19] Fulton R. W. Ridpath J. F. Confer A. W. Saliki J. T. Burge L. J. Payton M. E. (2003). Bovine viral diarrhoea virus antigenic diversity: impact on disease and vaccination programmes. Biologicals 31, 89–95. doi: 10.1016/S1045-1056(03)00021-6, PMID: 12770537

[ref20] Gao Y. Wang S. Du R. Wang Q. Sun C. Wang N.. (2011). Isolation and identification of a bovine viral diarrhea virus from sika deer in China. Virol. J. 8, 1–6. doi: 10.1186/1743-422X-8-83 21352530 PMC3052189

[ref21] Harms P. W. Frankel T. L. Moutafi M. Rao A. Rimm D. L. Taube J. M.. (2023). Multiplex immunohistochemistry and immunofluorescence: a practical update for pathologists. Mod. Pathol. 36:100197. doi: 10.1016/j.modpat.2023.100197, PMID: 37105494

[ref22] Heidari Z. Rezatofighi S. E. Rastegarzadeh S. (2021). Development and comparison of cross-linking and non-crosslinking probe-gold nanoparticle hybridization assays for direct detection of unamplified bovine viral diarrhea virus-RNA. BMC Biotechnol. 21, 1–12. doi: 10.1186/s12896-021-00691-w 33892712 PMC8063192

[ref23] Henrique M. Ramos-Júnior J. Flatschart R. Barroso S. Heinemann M. da Fonseca F.. (2023). Validation of a bovine viral diarrhea virus (BVDV) absolute quantification method by digital droplet PCR (ddPCR). J. Phys. 2606:012016. doi: 10.1088/1742-6596/2606/1/012016

[ref24] Hertig C. Pauli U. Zanoni R. Peterhans E. (1991). Detection of bovine viral diarrhea (BVD) virus using the polymerase chain reaction. Vet. Microbiol. 26, 65–76. doi: 10.1016/0378-1135(91)90042-E 1850892

[ref25] Hilbe M. Stalder H. Peterhans E. Haessig M. Nussbaumer M. Egli C.. (2007). Comparison of five diagnostic methods for detecting bovine viral diarrhea virus infection in calves. J. Vet. Diagn. Invest. 19, 28–34. doi: 10.1177/104063870701900105, PMID: 17459829

[ref26] Hou P. Xu Y. Wang H. He H. (2020). Detection of bovine viral diarrhea virus genotype 1 in aerosol by a real time RT-PCR assay. BMC Vet. Res. 16:114. doi: 10.1186/s12917-020-02330-6, PMID: 32295612 PMC7159024

[ref27] Hwang S. Lee W. Lee Y. (2023). Development of a nucleic acid detection method based on the CRISPR-Cas13 for point-of-care testing of bovine viral diarrhea virus-1b. J. Anim. Sci. Technol. doi: 10.5187/jast.2023.e77 PMC11331364 39165749

[ref28] Kim M. W. Lee D. Y. Cho C. H. Park C. Y. Ghosh S. Hyun M. S.. (2023). Sensitive detection of BVDV using gold nanoparticle-modified few-layer black phosphorus with affinity peptide-based electrochemical sensor. ACS Appl. Bio Mat. 6, 1621–1628. doi: 10.1021/acsabm.3c00045, PMID: 36972355

[ref29] Kim M. W. Park H.-J. Park C. Y. Kim J. H. Cho C. H. Park J. P.. (2020). Fabrication of a paper strip for facile and rapid detection of bovine viral diarrhea virus via signal enhancement by copper polyhedral nanoshells. RSC Adv. 10, 29759–29764. doi: 10.1039/D0RA03677C, PMID: 35518256 PMC9056175

[ref30] La Polla R. Testard M.-C. Garcia O. Goumaidi A. Legras-Lachuer C. de Saint-Vis B. (2022a). Involvement of the Wnt pathway in BVDV cytopathogenic strain replication in primary bovine cells. Virol. J. 19, 1–11. doi: 10.1186/s12985-022-01863-6 35986298 PMC9389679

[ref31] La Polla R. Testard M.-C. Goumaidi A. Chapot E. Legras-Lachuer C. de Saint-Vis B. (2022b). Identification of differentially expressed gene pathways between cytopathogenic and non-cytopathogenic BVDV-1 strains by analysis of the transcriptome of infected primary bovine cells. Virology 567, 34–46. doi: 10.1016/j.virol.2021.12.005, PMID: 34953294

[ref32] Letellier C. Kerkhofs P. Wellemans G. Vanopdenbosch E. (1999). Detection and genotyping of bovine diarrhea virus by reverse transcription-polymerase chain amplification of the 5′ untranslated region. Vet. Microbiol. 64, 155–167. doi: 10.1016/S0378-1135(98)00267-3, PMID: 10028170 PMC7117503

[ref33] Li J. Macdonald J. (2015). Advances in isothermal amplification: novel strategies inspired by biological processes. Biosens. Bioelectron. 64, 196–211. doi: 10.1016/j.bios.2014.08.069, PMID: 25218104

[ref34] Liang H. Geng J. Bai S. Aimuguri A. Gong Z. Feng R.. (2019). TaqMan real-time PCR for detecting bovine viral diarrhea virus. Pol. J. Vet. Sci. 22, 405–413. doi: 10.24425/pjvs.2019.129300, PMID: 31269348

[ref35] Lung O. Furukawa-Stoffer T. Burton Hughes K. Pasick J. King D. Hodko D. (2017). Multiplex RT-PCR and automated microarray for detection of eight bovine viruses. Transbound. Emerg. Dis. 64, 1929–1934. doi: 10.1111/tbed.12591, PMID: 27878975 PMC7169755

[ref36] Luo Y. Nartker S. Miller H. Hochhalter D. Wiederoder M. Wiederoder S.. (2010). Surface functionalization of electrospun nanofibers for detecting *E. coli* O157: H7 and BVDV cells in a direct-charge transfer biosensor. Biosens. Bioelectron. 26, 1612–1617. doi: 10.1016/j.bios.2010.08.028, PMID: 20833013

[ref37] Marzocca M. Seki C. Giambiagi S. Robiolo B. Schauer R. Santos M. D.. (2007). Truncated E2 of bovine viral diarrhea virus (BVDV) expressed in *Drosophila melanogaster* cells: a candidate antigen for a BVDV ELISA. J. Virol. Methods 144, 49–56. doi: 10.1016/j.jviromet.2007.03.023, PMID: 17512989

[ref38] Merwaiss F. Czibener C. Alvarez D. E. (2019). Cell-to-cell transmission is the main mechanism supporting bovine viral diarrhea virus spread in cell culture. J. Virol. 93:01776-01718. doi: 10.1128/JVI.01776-18 PMC6340029 30404802

[ref39] Metkar S. K. Girigoswami K. (2019). Diagnostic biosensors in medicine–a review. Biocatal. Agric. Biotechnol. 17, 271–283. doi: 10.1016/j.bcab.2018.11.029

[ref40] Mirosław P. Polak M. (2019). Increased genetic variation of bovine viral diarrhea virus in dairy cattle in Poland. BMC Vet. Res. 15, 1–12. doi: 10.1186/s12917-019-2029-z 31382966 PMC6683398

[ref41] Monteiro F. L. Cargnelutti J. F. Martins B. Noll J. G. Weiblen R. Flores E. F. (2019). Detection of bovine pestiviruses in sera of beef calves by a RT-PCR based on a newly designed set of pan–bovine pestivirus primers. J. Vet. Diagn. Invest. 31, 255–258. doi: 10.1177/1040638719826299, PMID: 30698509 PMC6838837

[ref42] Mucellini C. I. Silva Júnior J. V. J. de Oliveira P. S. B. Weiblen R. Flores E. F. (2023). Novel genomic targets for proper subtyping of bovine viral diarrhea virus 1 (BVDV-1) and BVDV-2. Virus Genes 59, 836–844. doi: 10.1007/s11262-023-02022-x, PMID: 37589803

[ref43] Munawar M. A. (2022). Critical insight into recombinase polymerase amplification technology. Expert. Rev. Mol. Diagn. 22, 725–737. doi: 10.1080/14737159.2022.2109964, PMID: 35950726

[ref44] Notomi T. Okayama H. Masubuchi H. Yonekawa T. Watanabe K. Amino N.. (2000). Loop-mediated isothermal amplification of DNA. Nucleic Acids Res. 28, 63e–e63. doi: 10.1093/nar/28.12.e63, PMID: 10871386 PMC102748

[ref45] Nugroho W. Silitonga R. J. P. Reichel M. P. Irianingsih S. H. Wicaksono M. S. (2022). The epidemiology and control of bovine viral diarrhoea virus in tropical Indonesian cattle. Pathogens 11:215. doi: 10.3390/pathogens11020215, PMID: 35215158 PMC8878523

[ref46] Odeón A. C. Leunda M. R. Faverín C. Boynak N. Vena M. Zabal O. (2009). *In vitro* amplification of BVDV field strains isolated in Argentina: effect of cell line and culture conditions. Rev. Argent. Microbiol. 41, 79–85. PMID: 19623896

[ref47] Oguejiofor C. F. Thomas C. Cheng Z. Wathes D. C. (2019). Mechanisms linking bovine viral diarrhea virus (BVDV) infection with infertility in cattle. Anim. Health Res. Rev. 20, 72–85. doi: 10.1017/S1466252319000057, PMID: 31895016

[ref48] Pang F. Long Q. (2023). Recent advances in diagnostic approaches for orf virus. Appl. Microbiol. Biotechnol. 107, 1515–1523. doi: 10.1007/s00253-023-12412-8, PMID: 36723701

[ref49] Pang F. Long Q. Wei M. (2023). Immune evasion strategies of bovine viral diarrhea virus. Front. Cell. Infect. Microbiol. 13:1282526. doi: 10.3389/fcimb.2023.1282526, PMID: 37900320 PMC10613064

[ref50] Piña R. Santos-Díaz A. I. Orta-Salazar E. Aguilar-Vazquez A. R. Mantellero C. A. Acosta-Galeana I.. (2022). Ten approaches that improve immunostaining: a review of the latest advances for the optimization of immunofluorescence. Int. J. Mol. Sci. 23:1426. doi: 10.3390/ijms23031426, PMID: 35163349 PMC8836139

[ref51] Qi S. Wo L. Sun C. Zhang J. Pang Q. Yin X. (2022). Host cell receptors implicated in the cellular tropism of BVDV. Viruses 14:2302. doi: 10.3390/v14102302, PMID: 36298858 PMC9607657

[ref52] Rivas J. Hasanaj A. Deblon C. Gisbert P. Garigliany M.-M. (2022). Genetic diversity of bovine viral diarrhea virus in cattle in France between 2018 and 2020. Front. Vet. Sci. 9:1028866. doi: 10.3389/fvets.2022.1028866, PMID: 36304414 PMC9593101

[ref53] Rodriguez A. Alonso-Morales R. A. Lassala A. Rangel P L. Ramírez-Andoney V. Gutierrez C. G. (2023). Development and validation of a pentaplex assay for the identification of antibodies against common viral diseases in cattle. Access Microbiol. 5, 000511–v000513. doi: 10.1099/acmi.0.000511.v3 PMC10634487 37970075

[ref54] Sandvik T. (2005). Selection and use of laboratory diagnostic assays in BVD control programmes. Prev. Vet. Med. 72, 3–16. doi: 10.1016/j.prevetmed.2005.08.015, PMID: 16168503

[ref55] Shapouri M. R. S. A. Mahmoodi P. Najafabadi M. G. Haji-Kolaei M. R. H. Choghakabodi P. M. Lotfi M.. (2022). A novel competitive ELISA for detection of antibodies against bovine viral diarrhea virus infection. Vet. Res. Forum 13, 403–407. doi: 10.30466/vrf.2021.521500.3135 36320291 PMC9548221

[ref56] Spetter M. J. Louge Uriarte E. L. Armendano J. I. Morrell E. L. Cantón G. J. Verna A. E.. (2020). Detection methods and characterization of bovine viral diarrhea virus in aborted fetuses and neonatal calves over a 22-year period. Braz. J. Microbiol. 51, 2077–2086. doi: 10.1007/s42770-020-00296-z, PMID: 32415638 PMC7688805

[ref57] Su A. Fu Y. Meens J. Yang W. Meng F. Herrler G.. (2021). Infection of polarized bovine respiratory epithelial cells by bovine viral diarrhea virus (BVDV). Virulence 12, 177–187. doi: 10.1080/21505594.2020.1854539, PMID: 33300445 PMC7801128

[ref58] Suda Y. Murakami S. Horimoto T. (2019). Bovine viral diarrhea virus non-structural protein NS4B induces autophagosomes in bovine kidney cells. Arch. Virol. 164, 255–260. doi: 10.1007/s00705-018-4045-x, PMID: 30259142

[ref59] Tabatabaei M. S. Ahmed M. (2022). “Enzyme-linked immunosorbent assay (ELISA)” in Cancer cell biology: Methods and protocols (Berlin: Springer), 115–134. 10.1007/978-1-0716-2376-3_10 35737237

[ref60] Tajbakhsh A. Rezatofighi E. Mirzadeh K. Pourmahdi M. (2017). A reverse transcriptase-loop mediated isothermal amplification assay (RT-LAMP) for rapid detection of bovine viral diarrhea virus 1 and 2. Archives of Razi Institute 72, 73–81. doi: 10.22092/ARI.2017.109836

[ref61] Tao J. Liao J. Wang Y. Zhang X. Wang J. Zhu G. (2013). Bovine viral diarrhea virus (BVDV) infections in pigs. Vet. Microbiol. 165, 185–189. doi: 10.1016/j.vetmic.2013.03.010 23587625

[ref62] Thür B. Zlinszky K. Ehrensperger F. (1996). Immunohistochemical detection of bovine viral diarrhea virus in skin biopsies: a reliable and fast diagnostic tool. J. Veterinary Med. Ser. B 43, 163–166. doi: 10.1111/j.1439-0450.1996.tb00301.x, PMID: 8928576

[ref63] Wang J. Chen K.-Y. Wang S.-H. Liu Y. Zhao Y.-Q. Yang L.. (2023). Effects of spatial expression of activating transcription factor 4 on the pathogenicity of two phenotypes of bovine viral diarrhea virus by regulating the endoplasmic reticulum-mediated autophagy process. Microbiol. Spectr. 11, e04225–e04222. doi: 10.1128/spectrum.04225-22 36939351 PMC10101076

[ref64] Weber M. N. Bauermann F. V. Gómez-Romero N. Herring A. D. Canal C. W. Neill J. D.. (2017). Variation in pestivirus growth in testicle primary cell culture is more dependent on the individual cell donor than cattle breed. Vet. Res. Commun. 41, 1–7. doi: 10.1007/s11259-016-9666-5, PMID: 27864728

[ref65] Wernike K. Beer M. (2024). Comparison of bovine viral diarrhea virus detection methods: results of an international proficiency trial. Vet. Microbiol. 290:109985. doi: 10.1016/j.vetmic.2024.109985, PMID: 38219410

[ref66] Workman A. M. Heaton M. P. Vander Ley B. L. Webster D. A. Sherry L. Bostrom J. R.. (2023). First gene-edited calf with reduced susceptibility to a major viral pathogen. PNAS Nexus 2:pgad125. doi: 10.1093/pnasnexus/pgad125, PMID: 37181049 PMC10167990

[ref67] Workman A. M. Heaton M. P. Webster D. A. Harhay G. P. Kalbfleisch T. S. Smith T. P.. (2021). Evaluating large spontaneous deletions in a bovine cell line selected for bovine viral diarrhea virus resistance. Viruses 13:2147. doi: 10.3390/v13112147, PMID: 34834954 PMC8622392

[ref68] Yang S. Wang Q.-Y. Tan B. Shi P.-F. Qiao L.-J. Li Z.-J.. (2022). A lateral flow dipstick combined with reverse transcription recombinase polymerase amplification for rapid and visual detection of the BVDV and BPIV3. J. Virol. Methods 299:114343. doi: 10.1016/j.jviromet.2021.114343, PMID: 34728269

[ref69] Yang N. Xu M. Ma Z. Li H. Song S. Gu X.. (2023). Detection of emerging HoBi-like Pestivirus (BVD-3) during an epidemiological investigation of bovine viral diarrhea virus in Xinjiang: a first-of-its-kind report. Front. Microbiol. 14:1222292. doi: 10.3389/fmicb.2023.1222292, PMID: 37492265 PMC10365292

[ref70] Yang G. Zhang J. Wang S. Wang J. Wang J. Zhu Y.. (2021). Gypenoside inhibits bovine viral diarrhea virus replication by interfering with viral attachment and internalization and activating apoptosis of infected cells. Viruses 13:1810. doi: 10.3390/v13091810 34578391 PMC8473207

[ref71] Yang N. Zhang J. Xu M. Yi J. Wang Z. Wang Y.. (2022). Virus-like particles vaccines based on glycoprotein E0 and E2 of bovine viral diarrhea virus induce humoral responses. Front. Microbiol. 13:1047001. doi: 10.3389/fmicb.2022.1047001, PMID: 36439839 PMC9687372

[ref72] Yao R. Xu Y. Wang L. Wang D. Ren L. Ren C.. (2021). CRISPR-Cas13a-based detection for bovine viral diarrhea virus. Front. Vet. Sci. 8:603919. doi: 10.3389/fvets.2021.603919, PMID: 34179152 PMC8219879

[ref73] Young N. J. Thomas C. J. Collins M. E. Brownlie J. (2006). Real-time RT-PCR detection of bovine viral Diarrhoea virus in whole blood using an external RNA reference. J. Virol. Methods 138, 218–222. doi: 10.1016/j.jviromet.2006.08.008, PMID: 17030066 PMC7112878

[ref74] Zhang X. Diraviyam T. Li X. Yao G. Michael A. (2016). Preparation of chicken IgY against recombinant E2 protein of bovine viral diarrhea virus (BVDV) and development of ELISA and ICA for BVDV detection. Biosci. Biotechnol. Biochem. 80, 2467–2472. doi: 10.1080/09168451.2016.1217144, PMID: 27484991

[ref75] Zhang X.-J. Han Q.-Y. Sun Y. Zhang X. Qiu H.-J. (2012). Development of a triplex TaqMan real-time RT-PCR assay for differential detection of wild-type and HCLV vaccine strains of classical swine fever virus and bovine viral diarrhea virus 1. Res. Vet. Sci. 92, 512–518. doi: 10.1016/j.rvsc.2011.03.029, PMID: 21536312

[ref76] Zhang N. Liu Z. Han Q. Qiu J. Chen J. Zhang G.. (2011). Development of one-step SYBR green real-time RT-PCR for quantifying bovine viral diarrhea virus type-1 and its comparison with conventional RT-PCR. Virol. J. 8, 1–8. doi: 10.1186/1743-422X-8-374 21798067 PMC3157457

[ref77] Zhang J. Wang W. Yang M. Lin J. Xue F. Zhu Y.. (2022). Development of a one-step multiplex real-time PCR assay for the detection of viral pathogens associated with the bovine respiratory disease complex. Front. Vet. Sci. 9:825257. doi: 10.3389/fvets.2022.825257, PMID: 35155658 PMC8825873

[ref78] Zhu J. Wang C. Zhang L. Zhu T. Li H. Wang Y.. (2022). Isolation of BVDV-1a, 1m, and 1v strains from diarrheal calf in China and identification of its genome sequence and cattle virulence. Front. Vet. Sci. 9:1008107. doi: 10.3389/fvets.2022.1008107, PMID: 36467650 PMC9709263

[ref79] Zimmer G. Wentink G. Bruschke C. Westenbrink F. Brinkhof J. De Goey I. (2002). Failure of foetal protection after vaccination against an experimental infection with bovine virus diarrhea virus. Vet. Microbiol. 89, 255–265. doi: 10.1016/S0378-1135(02)00203-1, PMID: 12383635

